# Identifying factors influencing local governments’ adoption of comprehensive smoke-free policies: an event history analysis based on panel data from 36 key cities in China (2013–2021)

**DOI:** 10.3389/fpubh.2024.1397803

**Published:** 2024-06-28

**Authors:** Wenting Feng, Binbin Qin, Xuezheng Jin, Shengyu Li

**Affiliations:** ^1^School of Journalism and Communication, Renmin University of China, Beijing, China; ^2^School of Public Administration and Policy, Renmin University of China, Beijing, China; ^3^Department of Health Communication, Chinese Center for Health Education, Beijing, China; ^4^School of Public Administration and Emergency Management, Jinan University, Guangzhou, China

**Keywords:** tobacco control, smoke-free policy, policy adoption, event history analysis, influencing factors

## Abstract

**Introduction:**

The issue of tobacco control remains a significant concern for public health worldwide. In recent years, remarkable progress has been made toward adopting smoke-free measures in indoor public places. Although China has yet to introduce a national regulation, specifically for smoke-free public places, more than a dozen cities have successively approved and implemented comprehensive smoke-free regulations. Different cities in China have diverse attitudes and behaviors toward smoke-free policies; however, the reasons for these policy differences and the influencing factors have not received sufficient attention and research.

**Methods:**

On the basis of the multiple streams framework, this study selects 36 key Chinese cities as research samples and uses a directed dyad-year event history analysis method to analyze the factors influencing the implementation of comprehensive smoke-free policies in cities.

**Results:**

Results show that the adoption of such policies is positively influenced by scientific evidence, focal events, media coverage, institutional foundations, economic comparisons, and the influence of health departments and of tobacco control groups. By contrast, policy adoption is negatively affected by the differences in administrative levels, central policy signals, and the influence of the tobacco industry.

**Discussion:**

This study contributes to understanding the internal logic behind local governments’ adoption of comprehensive smoke-free policies, offering insights for further advocacy at the city and national levels in China and providing experiences that can promote the global tobacco control movement.

## 1 Introduction

Tobacco control is a global public health issue of great concern. Promoting smoke-free measures in indoor public places is one of the core tobacco control strategies continuously advocated by the World Health Organization’s (WHO’s) Framework Convention on Tobacco Control (FCTC). To date, 74 countries have implemented policies that completely ban smoking in indoor public places, workplaces, and public transport, up from only 10 in 2007 and covering 2.1 billion people (1). As the largest tobacco producer and consumer in the world, the issue of smoking in public places in China is not optimistic, with approximately 740 million non-smokers suffering from secondhand smoke exposure (2), and a secondhand smoke exposure rate among non-smokers of 68.1%, leading to over 100,000 deaths annually (3). Despite joining the FCTC many years ago, China still does not have a national law that prohibits smoking in public places. With national legislation in a deadlock, tobacco control advocates have turned to local governments for breakthroughs. In recent years, a few cities in China have introduced smoke-free legislation, meeting the WHO’s best-practice requirements for a comprehensive smoke-free policy.

Public policy is the most effective way to address the tobacco epidemic (4). The case of China shows that different cities have diverse attitudes and behaviors toward smoke-free policies. Thus, what causes these differences in policies among cities? What factors influence the performance of cities in adopting smoke-free policies in public places? Previous public health research on this topic has mostly focused on the analysis of secondhand smoke exposure monitoring data (5), and the analysis of policies has mainly been the evaluation of the effects of policy implementation (6), lacking an explanation from the perspective of policy formation as to why different administrative authorities have variations in the adoption of tobacco control policies.

The field of tobacco control policy serves as a critical domain for generating theoretical knowledge about the policy process, contributing rich and in-depth empirical material on policy diffusion, policy learning, policy beliefs, and other research topics (7). Research in this area involves a number of countries and regions, including developed countries, such as the United States, Canada, Japan, and Australia (8–10), as well as developing countries, such as Uruguay, Indonesia, Nigeria, and Bangladesh (11–13). As a nation severely affected by tobacco use, the issue of tobacco control in China has also attracted the attention of scholars. Scholars have conducted extensive and in-depth research on smoking behavior at both the micro level of individuals (14) and the meso level of groups (15), providing many insightful ideas for reducing tobacco harm. However, few scholars have focused on the impact of government actions on tobacco control at the macro level. The strategies and experiences of Chinese policymakers in formulating tobacco control policies have not received enough attention from public health advocates and public policy researchers.

Therefore, this study attempts to introduce the perspective of public policy process theory to conduct an in-depth analysis of the factors influencing local governments’ enactment of comprehensive smoke-free policies. The primary question it aims to address is: What factors have influenced the adoption of smoke-free policies by local governments in China, and how?

Utilizing the multiple streams framework as its theoretical foundation, this study considers the characteristics of China’s political and administrative structures. It adapts and extends the framework to suit the specific circumstances surrounding China’s local tobacco control policies, thereby creating an analytical framework to explore what influences local governments to implement comprehensive smoke-free policies and formulating relevant hypotheses accordingly. Furthermore, this study tests the proposed hypotheses using the directed dyad-year event history analysis (EHA) method. Panel data are collected from 36 key cities in China’s provincial capitals and above from 2013 to 2021. Using these data, regression analyses are conducted to analyze the factors influencing the adoption of comprehensive smoke-free policies in cities using a discrete-time logit model.

Since 2016, with the official adoption of the “Healthy China” national strategy, China has emphasized the formulation of public policies that embody health-centric principles as an important way to realizing this national strategy (16). In this study, a representative health policy, the comprehensive smoke-free policy, is selected, and the factors influencing the implementation of the policy are analyzed. Results of the study will be useful for the promotion of tobacco control in China, as well as globally. The study also provides some guidance on how to improve the level and quality of social policy, especially health policy formulation. It also has a positive effect on improving the level and capacity of local government governance.

## 2 Literature review and analytical framework

In addressing global public health challenges, the issue of tobacco control has attracted extensive attention from scholars across different fields worldwide. Overall, research on the adoption of smoking control policies has generally reached the following consensus. First, it emphasizes the contextuality of policy formulation. Smoking behavior is influenced by a complex interplay of historical, social, cultural, psychological, and physiological factors, and the design of tobacco control programs needs to consider social and cultural contexts (17). Second, increasing attention has been paid to the structural socioeconomic and political factors behind the tobacco epidemic (18). In recent years, the effects of political factors on the adoption of public health policies have been particularly emphasized. For example, studies have found that community coalitions can form under various sociopolitical contexts, thereby promoting cooperation among multiple departments of local governments and facilitating the adoption of public health policies (19). Third, the interaction among various policy actors and their effects on policies have garnered considerable interest. For example, scholars have highlighted the role of the media, as well as local and international tobacco control groups, in influencing Japan’s tobacco control policies (20). By contrast, the tobacco industry has been particularly dominant in some small island developing countries (11). These studies have provided insightful research perspectives and analytical frameworks for examining the adoption of tobacco control policies in China. However, given the uniqueness of political and administrative systems, the analysis of China’s tobacco control policies requires an inclusive theoretical framework and elements of interpretation based on the Chinese context.

**Figure 1:**
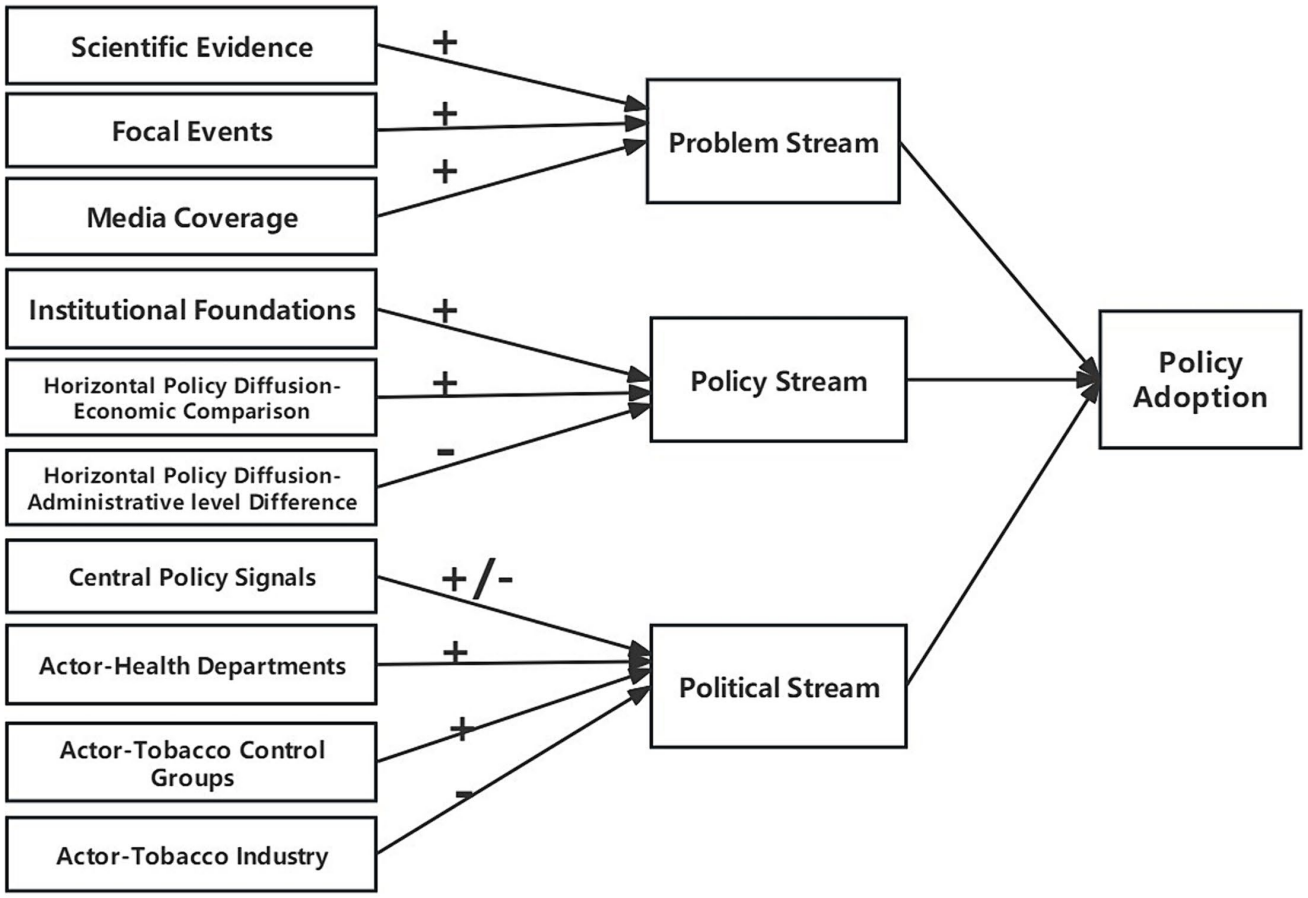
Analytical framework for factors influencing local governments’ adoption of comprehensive smoke-free policies.

This study applies the multiple streams framework as its theoretical basis. As a classic theoretical framework for the public policy formulation process, this framework boasts significant explanatory power and applicability over the past few decades (21). In terms of application areas, the framework has been used for policy process analysis in more than 20 fields, including health, environment, governance, education, and welfare, involving levels such as international, national, and local policies across more than 60 countries with different political backgrounds (22). The multiple streams framework proposes that public policy occurs in a field full of ambiguity, contingency, and uncertainty, where the policy agenda and alternatives are the result of the combined action of problem, policy, and political streams (23). On the basis of this framework, this study constructs an analytical framework tailored to the specific context of China’s tobacco control policy (see Figure 1), proposes corresponding hypotheses, and tests them in subsequent empirical analyses.

### 2.1 Problem stream-related hypotheses

The problem stream refers to how problems are recognized and defined, and significant events or crises, indicators, and feedback all potentially trigger policymakers’ attention to problems. Currently, tobacco control has yet to become a central task for governments at all levels in China (24); thus, the space for tobacco control policies to enter the decision-making agenda of local governments is extremely limited. Local governments’ attention to tobacco control in public places comes from three main sources: scientific evidence, focal events, and news reports.

First, scientific evidence is one of the core pieces commonly used in public health (25). Internationally, the ever-emerging scientific evidence demonstrating the strong association between smoking (including passive smoking) and diseases has led to the recognition of tobacco control as a public health issue by governments, which, in turn, have introduced strict policy measures to intervene and control smoking behavior (26). Therefore, local government policymakers, when presented with ample scientific evidence related to smoking and health issues in their region, are more likely to focus on tobacco control issues and thus formulate corresponding policies. Second, events are an important factor in the study of the public policy process, including sudden, unexpected events and planned, foreseeable ones (27). The practice of tobacco control in China shows that when a specific city plans to host major international activities, such as sporting events or exhibitions, it will bring a valuable policy window for tobacco control policies in public places. Local governments are more likely to consider the effects of public place smoking policies on the city’s international image during this period (28). Lastly, agenda setting is considered a key link in the formation of public policy, and the mainstream media, by reporting on existing issues, influence decision makers’ perceptions of the importance or severity of problems. In this sense, “deciding which issues will become policy issues is even more important than deciding which will become solutions” (29).

Accordingly, three hypotheses related to the problem stream are proposed:

*H1*: Cities with more comprehensive scientific evidence are more likely to introduce comprehensive smoke-free environment policies.

*H2*: City governments in the period of major international activities are more likely to introduce comprehensive smoke-free environment policies.

*H3*: Cities with more media reports on tobacco control are more likely to introduce comprehensive smoke-free environment policies.

### 2.2 Policy stream-related hypotheses

The policy stream refers to the process through which policy proposals or advocacies are proposed. For policymakers, judgment on proposals comes partly from internal existing practices, that is, the existing institutional foundation, and partly from the experiences of practices in other areas, namely, the influence of horizontal policy diffusion.

First, for local governments, adopting an innovative policy is a risky act, and adopting a policy with a certain institutional basis or better policy compatibility can reduce the potential political, economic, and social risks after policy implementation. The stronger the policy compatibility is, the higher the likelihood of the government adopting that policy will be (30). For example, the more tobacco control policies a local government has issued, the stronger the compatibility of a comprehensive smoke-free policy with existing policies in terms of ideology and action, and the higher the likelihood of it being adopted. Second, in addition to the internal policy community, the actions of external policy communities in other regions or countries also influence the government’s evaluation or judgment of the policy stream (31). When a policy is successfully adopted in a certain region, indicating that the policy stream is mature, other areas are more likely to learn or imitate it. Previous research has shown that governments with higher levels of economic development often have better governance levels and policy performance, serving as a model for other regions (32). Third, local governments, when facing uncertainty of outcomes and complexity of the environment, tend to imitate peer governments with similar circumstances (33). Therefore, this study considers the level of economic development and city level as the main influencing variables for the horizontal diffusion of policies.

Accordingly, three hypotheses related to the policy stream are proposed:

*H4*: Cities with a greater number of existing smoking control policies are more likely to introduce comprehensive smoke-free environment policies.

*H5*: The likelihood of a city implementing a comprehensive smoke-free policy increases after economically more developed cities adopt such policies.

*H6*: The likelihood of a city adopting a comprehensive smoke-free policy increases after other cities with a similar administrative level implement such policies.

### 2.3 Political stream-related hypotheses

The political stream refers to the political and cultural context that influences the agenda or outcomes, including public sentiment, competition among interest groups, election results, political party ideologies, and changes in government. For local governments in China, on the one hand, policy signals from the central government constitute an important political context for decision making; on the other hand, distinguishing the competition among interest groups into different actors is more fitting.

As noted earlier, the attention resources allocated by the Chinese central government to the tobacco control subsystem are relatively limited, without the presence of hard compulsory pressure. However, the central government continues to advocate by releasing positive policy signals, such as issuing guidelines and planning outlines related to the field of tobacco control. When local city governments observe continuous positive tobacco control policy signals from the central government, they are more likely to introduce stricter comprehensive smoke-free policies to express their support for the central government. Research has also shown that there might be a more complex relationship between central policy signals and local government actions (34). When the central government has already clearly sent policy signals, local government’s policy adoption might lose a degree of innovation and pioneering spirit, thereby weakening the local government’s motivation to adopt policies.

This condition leads to two competing hypotheses about central policy signaling:

*H7a*: The stronger the central policy signals is, the higher the likelihood of cities introducing comprehensive smoke-free environment policies will be.

*H7b*: The weaker the central policy signals is, the higher the likelihood of cities introducing comprehensive smoke-free environment policies will be.

Interactions among specific actors in a particular field are more important for understanding policy change than macro factors, such as economic development and social movements (35). The influence of different actors on policy varies significantly. The three main core actors that influence tobacco control policy at the local level in China are the health departments, tobacco control groups, and the tobacco industry. First, health departments are the primary responsible departments for tobacco control work, participating throughout and regularly in tobacco control policy issues, influencing all stages of policy development. Second, tobacco control groups refer to social organizations involved in advocating tobacco control policies, including Chinese grassroots and international tobacco control organizations. Notably, China’s tobacco control process is deeply influenced by international tobacco control organizations. Particularly, the technical support and financial assistance received from international tobacco control organizations since the 1980s has had a profound influence on China’s tobacco control progress. Third, the obstruction of the tobacco control process by the tobacco industry is a common occurrence in all countries worldwide (36). The tobacco monopoly system implemented in China endows the tobacco industry with the dual identity of government manager and industry owner, making it the core actor hindering the tobacco control process. The tobacco industry exerts policy influence by providing financial resources to local governments. The more financial resources the tobacco industry supplies to a city, the greater its influence, and the lower the likelihood of the government adopting strict tobacco control policies.

Thus, three research hypotheses regarding actors are proposed:

*H8*: The greater the influence of health departments is, the higher the likelihood of cities introducing comprehensive smoke-free environment policies will be.

*H9*: The greater the influence of tobacco control groups is, the higher the likelihood of cities introducing comprehensive smoke-free environment policies will be.

*H10*: The greater the influence of the tobacco industry is, the lower the likelihood of cities introducing comprehensive smoke-free environment policies will be.

## 3 Methods

### 3.1 Sample selection

The adoption of comprehensive smoke-free policies in China demonstrates a distinctive “local initiative” characteristic (37). Currently, a nationwide comprehensive smoke-free policy has yet to be implemented, whereas at the local level, some cities have already achieved the comprehensive smoke-free regulations stipulated by the WHO. Given that the tobacco control performance of provincial capitals and above has an exemplary effect on the region and even the whole country and that they have local legislative power to introduce local tobacco control laws and regulations, this study selects 36 cities, including provincial capitals and above, as research samples. This specifically includes 4 municipalities directly under the central government, 5 subprovincial cities, and 27 provincial capitals.

### 3.2 Statistical methods

This study employs the directed dyad-year EHA method to analyze panel data of 36 key cities. EHA, also known as survival analysis, is highly applicable for exploring the factors influencing the probability of an event’s occurrence and has become a mainstream analytical method in policy innovation diffusion research (38). In recent years, the directed dyad-year EHA has gradually replaced the traditional EHA method and has gained increasing attention and applications (39). The directed dyad-year EHA can provide further insights into the micro-diffusion mechanism among different subjects and deepen policy diffusion research (40). Accordingly, this study uses the directed dyad-year EHA to investigate the diffusion of comprehensive smoke-free environment policies across 36 key cities. The research conducts regression analysis on the factors influencing the introduction of comprehensive smoke-free policies in cities using the discrete-time logit model through Stata 16.0 statistical software.

### 3.3 Measurement and data sources

The data collected for this study spans from 2013 to 2021, covering the panel data of 36 cities from the introduction of the first city-wide comprehensive smoke-free policy in Qingdao in 2013 until 2021. Policy data primarily comes from the Chinese Laws and Regulations Database (BEIDA FABAO), which is one of the most comprehensive legal databases in China. Other variable data are mainly from the official statistical yearbooks of the cities and the national industry-specific yearbooks.

According to the coding rules of directed dyad-year EHA (41), this method assumes a sequential order of policy diffusion among regions, where *i* represents the potential policy adopter or learner; and *j* is the potential policy pioneer or learner, who has adopted the policy earlier than *i*. The dependent variable *policy adoption* is a binary dummy variable. When city *i* is paired with city *j*, if city *i* adopts a comprehensive smoke-free policy in year *t*, and city *j* had already adopted the policy in year *t* − 1 or earlier, then the policy adoption (pairing) for city *i* in year *t* is valued at 1, otherwise it is 0. After city *i* adopts a comprehensive smoke-free environment policy in year *t*, its observations from year *t* + 1 onwards are excluded; thus, the sample is subject to right-censoring. Through pairing, an unbalanced panel data set is formed, with the number of pairings or observations being 1,575.

Table 1 presents the detailed descriptions and measurement methods of the variables. The study uses economically relevant indicators to measure the influence of the health sector and the tobacco industry within the government system, that is, the share of health and wellness expenditures as a percentage of the city’s general fund expenditures and the share of taxes paid by the city’s tobacco industry as a percentage of the city’s total tax revenues. Previous empirical research on the diffusion of tobacco control policy innovations has shown that local governments with a high share of health and wellness fiscal expenditures are likely to adopt restrictive smoking policies to reduce tobacco-related healthcare costs (42). In addition, the nature of the government’s allocation of public funds is the government’s goal orientation and power structure (43). Although the tobacco industry’s contribution to local finances is considered to be the fundamental reason for influencing government policy (44), the study uses tobacco tax payments as a measure of the tobacco industry’s influence.

**Table 1:** Variables and measurement.

Variable name	Measurement description
Dependent variable (Y)	
Policy adoption	A binary variable assigned 1 if city *i* adopts a comprehensive smoke-free policy in year *t* and city *j* had already adopted the policy in year *t* − 1 or earlier; otherwise, it is 0.
Independent variables (X)	
Scientific evidence	Assigned 1 if city *i* conducted surveys on tobacco prevalence or secondhand smoke exposure in year *t* − 1; otherwise, it is 0.
Focal events	Assigned 1 for the year and the two years prior to when city *i* hosts significant international events; otherwise, it is 0.
Media coverage	The number of news reports related to smoking control in city *i* in the given year (in thousands).
Policy foundations	The number of smoking control policies in public places enacted in city *i* by the end of the previous year.
Horizontal policy diffusion-economic comparison	Assigned 1 if city *j*’s *per capita* GDP in year *t* − 1 is higher than that of city *i*; otherwise, it is 0.
Horizontal policy diffusion-administrative level difference	Municipalities directly under the central government are scored as 3, subprovincial cities as 2, and other non-subprovincial capital cities as 1. The score for this item is the difference between the administrative level score of cities *j* and *i*.
Central policy signals	The number of policies issuing public smoking control by the central government in year *t* − 1.
Influence of health departments	The proportion of healthcare expenditure to the general budget expenditure of city i in year *t* − 1 (in %).
Influence of tobacco control groups	Encoded as 1 if city *i* was selected for the smoke-free city legislation project in year *t* − 1; otherwise, it is 0.
Influence of the tobacco industry	The percentage of total tobacco tax revenue to the total tax revenue of city *i*/province in year *t* − 1 (in %).

To measure the pressure of the central policy, the study examines the tobacco control policies on public places issued at the national level from 2011 to 2021, obtaining a total of 37 policy texts (see Supplementary Table S1). These texts include different policy categories, such as departmental normative documents, departmental regulations, State Council normative documents, administrative regulations, and legal working documents, all of which have made relevant provisions on “smoking behavior in public places.” The policy pressure is the number of policy documents on tobacco control in public places issued in the previous year. Given that the effects of policies may have a certain time lag, the study further uses *t* − 2 data for the robustness test of the central policy pressure.

## 4 Results

### 4.1 Comprehensive smoke-free policies in provincial capitals and above in China

By using the keywords “city name,” “public places,” and “smoking,” this study conducted a search and review of the smoke-free policies texts issued by various cities through the Chinese Laws and Regulations Database and the official websites of each city government. As of December 31, 2021, a total of 107 regulations or rules related to smoke-free policies were obtained from 36 cities (including amendments). The policy texts were evaluated on an article-by-article basis with reference to Article 8 of the WHO FCTC and its implementation guidelines for a smoke-free environment, and cities that achieve a smoke-free environment should meet the following criteria: “Smoke-free places should cover all indoor public spaces. Smoke-free places should cover all indoor public places, indoor workplaces, and public transportation,” or at least the eight categories recommended by the WHO (i.e., healthcare facilities, schools, universities, government facilities, offices, restaurants, bars and other entertainment venues, and public transport) if they are enumerated as smoke-free places (45). The policy texts must not allow designated smoking areas, and if a transition period is set, then a specific end date must be clearly stated (46). The specific assessment results for each city are provided in Supplementary Table S2. Ultimately, out of China’s 36 provincial capitals and cities above, 13 cities have enacted comprehensive smoke-free policies in public places, namely, Qingdao, Shenzhen, Lanzhou, Beijing, Nanning, Shanghai, Changchun, Xi’an, Hangzhou, Wuhan, Harbin, Zhengzhou, and Xining.

### 4.2 Descriptive statistics

Table 2 presents the descriptive statistics for the main variables of this study.

**Table 2:** Descriptive statistical results of main variables.

Variable	Observation	Mean	Standard Deviation	Minimum	Maximum	VIF
Policy adoption	1,575	0.045	0.208	0	1	/
Scientific evidence	1,575	0.335	0.472	0	1	1.14
Focal events	1,575	0.227	0.419	0	1	1.23
Media coverage	1,575	1.407	1.576	0.014	17.353	1.65
Policy foundations	1,575	6.524	3.703	0	17	1.35
Horizontal policy diffusion-economic comparison	1,575	0.62	0.486	0	1	1.37
Horizontal policy diffusion-administrative level difference	1,575	0.402	0.943	−2	2	1.66
Central policy signals	1,575	3.326	1.973	1	6	1.11
Influence of health departments	1,575	7.217	1.616	2.77	11.750	1.05
Influence of tobacco control groups	1,575	0.208	0.406	0	1	1.40
Influence of the tobacco industry	1,575	6.939	7.139	0.454	43.447	1.14

The mean value of the dependent variable, policy adoption, is 0.045, indicating a 4.5% probability of policy adoption occurrence within the observed 1,575 samples. In terms of the problem stream-related variables, 33.5% of the samples conducted scientific surveys related to tobacco prevalence, highlighting the attention cities pay to scientific evidence in the policy process. The probability of focal events, namely, major international activities, is 22.7%, mainly involving cities of Beijing, Shanghai, Nanning, Hangzhou, Shenzhen, Nanjing, Qingdao, Tianjin, and Wuhan. A significant variation exists in tobacco control media coverage among the observed cities, with a maximum value of 17.353 (thousand articles) and a minimum value of just 0.014 (thousand articles). In terms of variables related to the policy stream, the institutional foundation varies among cities. Fuzhou, Changsha, and Taiyuan were later in issuing policy texts related to smoking control in public places, with these cities issuing relevant policy texts in 2015, 2018, and 2016, respectively. In terms of the variables related to political stream, the central policy signal values range from a maximum of 6 to a minimum of 1, indicating significant differences in the number of tobacco control policies issued at the central level. The maximum value for the tobacco industry influence indicator is 43.447, representing the percentage of tobacco taxes in Kunming’s total tax revenue in 2016, whereas the minimum value is for Dalian in 2014, where tobacco industry taxes accounted for only 0.454% of the city’s total tax revenue.

Table 2 also reports the variance inflation factor (VIF) for each variable, all below 10, with the highest at 1.66, suggesting a low likelihood of multicollinearity among the variables and that a strong degree of independence exists among the variables.

### 4.3 Logit regression model results

The study incrementally incorporated three sets of independent variables into the models for analysis. Models 1–3 are single-dimensional models, Models 4–6 are two-dimensional combination models, and Model 7 is a full model that incorporates all explanatory variables into the regression analysis. The results of the logit regression models are presented in Table 3.

**Table 3:** Results of the logit regression model.

Variable	Model 1	Model 2	Model 3	Model 4	Model 5	Model 6	Model 7
	Regression coefficient	Regression coefficient	Regression coefficient	Regression coefficient	Regression coefficient	Regression coefficient	Regression coefficient
Scientific evidence	2.998^***^ (0.464)			3.499^***^ (0.564)	3.865^***^ (0.816)		4.174^***^ (0.945)
Focal events	2.451^***^ (0.424)			2.782^***^ (0.480)	3.768^***^ (0.844)		3.720^***^ (1.295)
Media coverage	1.024^***^ (0.148)			0.960^***^ (0.147)	1.153^***^ (0.233)		1.260^***^ (0.330)
Policy foundations		0.155^***^ (0.041)		0.241^***^ (0.075)		0.139^***^ (0.053)	0.621^***^ (0.196)
Economic comparison		0.076 (0.327)		0.527 (0.505)		0.233 (0.404)	1.363^*^ (0.755)
Administrative level difference		−0.215 (0.189)		−0.143 (0.300)		−0.240 (0.241)	−0.940^**^ (0.409)
Central policy signals			−0.326^***^ (0.089)		−0.401^***^ (0.142)	−0.341^***^ (0.092)	−0.593^***^ (0.183)
Influence of health departments			−0.356^***^ (0.115)		0.875^***^ (0.325)	−0.393^***^ (0.120)	1.364^***^ (0.479)
Influence of tobacco control groups			4.012^***^ (0.395)		4.709^***^ (0.970)	3.930^***^ (0.391)	3.743^***^ (0.762)
Influence of the tobacco industry			−0.081^***^ (0.031)		−0.507^***^ (0.160)	−0.042 (0.032)	−0.463^**^ (0.192)
Observation	1,575	1,575	1,575	1,575	1,575	1,575	1,575
Pseudo *R*-squared	0.576	0.121	0.370	0.600	0.709	0.384	0.748
Log lik.	−122.804	−254.320	−182.478	−115.893	−84.158	−178.318	−72.936
Chi-squared	333.246^***^	70.215^***^	213.342^***^	347.070^***^	410.539^***^	222.220^***^	432.984^***^

Standard errors are in parentheses; *p < 0.1, ^**^p < 0.05, ^***^p < 0.01.

The pseudo *R*^2^ represents the model fit. All models are significant at the 0.01 level. Model 7 has the highest pseudo *R*^2^ at 0.748, indicating that it can explain 74.8% of the variance in the dependent variable. Among the single variable models, Model 1 has the highest pseudo *R*^2^, suggesting the problem stream has the strongest explanatory power for the dependent variable, followed by the political and policy streams. The full model has the highest fit among all models, indicating that policy adoption results from the interaction of multiple variables.

For variables related to the problem stream, scientific evidence, focal events, and media coverage significantly influence the adoption of comprehensive smoke-free policies in Models 1, 4, 5, and 7, all with positive effects, and H1, H2, and H3 pass the significance test at the 0.01 level. Specifically, with other variables controlled, the more comprehensive the scientific evidence is, the city being in a major international event cycle, and the more tobacco control media coverage is, the more likely the city will introduce a comprehensive smoke-free policy.

For the policy stream-related variables, institutional foundation passes the significance test at the 0.01 level in Models 2, 4, 6, and 7, whereas economic comparison and administrative level differences only pass in Model 7. Institutional foundation and economic comparison have positive effects, which indicates that the more smoking-related policies a city has issued and the higher the economic level of other cities that have adopted comprehensive smoke-free policies, the more inclined the city will be to introduce such policies. Administrative-level differences have negative effects, indicating that the smaller the difference in administrative levels between cities is, the more likely imitation behavior will occur. That is, if other cities of similar administrative level introduce comprehensive smoke-free policies, then the city is more likely to adopt such policies. H4, H5, and H6 pass the significance test at the 0.01, 0.1, and 0.05 levels, respectively, in Model 7.

Regarding variables related to the political stream, central policy signals pass the significance test at the 0.01 level in Models 3, 5, 6, and 7, with a negative correlation. This result indicates that the stronger the policy signals in the tobacco control field released by the central government are, the less likely the local governments will adopt comprehensive smoke-free policies. Actor influence shows that the health departments and tobacco control groups have significantly positive effects on policy adoption, whereas the tobacco industry’s influence has a significantly negative effect. This result indicates that actions by the health system and tobacco control groups promote local policy adoption of comprehensive smoke-free policies, whereas the tobacco system hinders it. In Model 7, H8, H9, and H10 pass the significance test at the 0.01, 0.01, and 0.05 levels, respectively.

Model 7 further analyzes the effects of various variables on policy adoption. Scientific evidence, the influence of tobacco control groups, and focal events are key to policy adoption, with coefficients in Model 7 of 4.174, 3.743, and 3.720 and odds ratios of 64.97, 42.22, and 41.26, respectively. Media coverage, institutional foundation, economic comparison, and the influence of health departments have odds ratios of 3.52, 1.86, 3.90, and 3.91, respectively. This result indicates that for every additional thousand articles of tobacco control media coverage, the odds of a city introducing a comprehensive smoke-free policy increase by 3.52 times; for every additional policy related to smoking control, the odds increase by 86%; if higher GDP level cities have adopted comprehensive smoke-free policies, the odds increase by 2.9 times; and for every percentage increase in public health spending, the odds increase by 2.91 times. Administrative level differences, central policy signals, and tobacco industry influence negatively affect policy adoption, with odds ratios of 0.38, 0.55, and 0.63, respectively. This outcome implies that for every unit increase in city level difference, the odds decrease by 62%; for every unit increase in central policy signal strength, the odds decrease by 45%; and for every percentage increase in tobacco industry tax contribution, the odds decrease by 37%.

### 4.4 Robustness test

Given that the dependent variable data in this study are unbalanced, with the occurrence probability of policy adoption being only 4.5%, there might be a rare events bias. Therefore, the study considers using a relogit model, which is suitable for rare events data, to conduct a robustness test (47). The relogit model regression results (see Table 4) indicate that all 10 independent variables have significant effects on the dependent variable, and the conclusions are consistent with those from the logit model regression results. This finding demonstrates that the study’s conclusions exhibit strong robustness.

**Table 4:** Results of relogit model regression.

Variable	Relogit
Scientific evidence	3.359^***^ (3.798)
Focal events	2.007^***^ (1.532)
Media coverage	1.295^**^ (0.517)
Policy foundations	0.698^***^ (0.192)
Horizontal policy diffusion-economic comparison	1.338^**^ (0.399)
Horizontal policy diffusion-administrative level difference	−0.839^**^ (0.350)
Central policy signals	−0.466^***^ (0.142)
Influence of health departments	1.381^***^ (0.489)
Influence of tobacco control groups	3.664^***^ (0.275)
Influence of the tobacco industry	−0.461^*^ (0.252)

***, **, and * denote significance levels at 1, 5, and 10%, respectively. Standard errors are in parentheses.

Given multi-level governance pattern, there may be delays in the transmission of central policy to local governments. In case there may be a lag effect of central policy, we conduct a lag analysis in the statistical model, using *t* − 2 data for the central policy signals variable in the logit analysis. The results (see Table 5) show that the *t* − 2 central policy signal variable is not significant. When *t* − 1 and *t* − 2 data are included in the model simultaneously, the results shows a negative correlation for *t* − 1, while *t* − 2 remains insignificant. These results are consistent with the baseline logit model.

**Table 5:** Results of central policy signals’ lag analysis.

Variable	Model 8	Model 9
Scientific evidence	3.688^***^ (0.725)	3.989^***^ (0.923)
Focal events	2.436^***^ (0.584)	2.603^**^ (1.288)
Media coverage	0.931^***^ (0.184)	0.728^***^ (0.352)
Policy foundations	0.531^***^ (0.172)	0.607^***^ (0.195)
Horizontal policy diffusion-economic comparison	0.977 (0.710)	1.341^*^ (0.762)
Horizontal policy diffusion-administrative level difference	−0.425 (0.368)	−0.897^**^ (0.418)
Central policy signals (*t* − 1)		−0.588^***^ (0.183)
Central policy signals (*t* − 2)	0.132 (0.156)	0.099 (0.185)
Influence of health departments	0.890^**^ (0.348)	1.264^**^ (0.497)
Influence of tobacco control groups	2.727^***^ (0.518)	3.741^***^ (0.777)
Influence of the tobacco industry	−0.302^**^ (0.136)	−0.474^**^ (0.199)

***, **, and * denote significance levels at 1, 5, and 10%, respectively. Standard errors are in parentheses.

## 5 Discussion

On the basis of the specific context of tobacco control policy formulation in China, this study proposes an analytical framework of the factors influencing the adoption of comprehensive smoke-free policies by local governments by applying the multiple streams framework, which is a classic theory in policy process research. It then empirically tests this framework using the EHA method. Statistical results show that all 10 core independent variables significantly influence the adoption of comprehensive smoke-free policies in cities, supporting the related hypotheses. Specifically, scientific evidence, focal events, media coverage, institutional foundation, economic comparisons, and the influence of health departments and tobacco control groups all positively affect policy adoption, whereas differences in administrative levels, central policy signals, and the influence of the tobacco industry have negative effects.

Scientific evidence, focal events, and media coverage constitute the problem stream for smoke-free policies, shaping policymakers’ perception of the severity of public place smoking issues. This study validates the successful experience of tobacco control policy making abroad that the future of developing smoke-free policies depends on reliable scientific data (48). Scientific data provide strong scientific support for decision makers to focus on public place smoking issues. Before initiating smoke-free policy formulation, conducting scientific tobacco prevalence surveys and collecting local tobacco harm evidence in the city become prerequisites for successful policy advocacy. Moreover, linking scientific evidence with political backgrounds and utilizing the “spillover effect” of other issues can effectively increase attention to the issue. For example, when preparing evidence for smoke-free environment policy, the health department of Chongqing paid particular attention to the negative effect of smoking on the poor, responding to China’s political goal of poverty alleviation. Focal events, mainly planned and foreseeable events, create an opportunity for cities to introduce smoke-free policies and opens a “policy window,” a feature that is particularly evident in the early stages of smoke-free advocacy (49). Cities such as Beijing, Shanghai, Hangzhou, Wuhan, and Nanning all took advantage of this critical timing to promote local smoke-free legislation. Given that major international events are predictable, tobacco control advocates should emphasize and take advantage of this opportunity to push comprehensive tobacco control policies onto government agendas. Media coverage shows a strong positive correlation with local governments adopting comprehensive smoke-free policies. Successful tobacco control advocacy is a process of broad expression and gaining acceptance by multiple stakeholders, especially tobacco control alliances, where mainstream media significantly becomes a conduit and platform for advocacy “upwards” and “downwards” (50). Following communication laws, accumulating, excavating, and releasing the public opinion momentum of macro social contexts and public issues remain an important strategy that should be adhered to and improved in future tobacco control communication.

Institutional foundation and horizontal policy diffusion significantly influence decision makers’ consensus on policy proposals. The existing institutional foundation significantly affects local governments’ adoption of comprehensive smoke-free policies. In China’s tobacco control practices, many cities have gradually aligned their public place smoking control laws and regulations with the convention requirements through multiple revisions, ultimately facilitating the introduction of comprehensive smoke-free policies. In view of the continuity and gradual nature of China’s public policy formulation (51), policy introductions often undergo minor modifications based on existing foundations, presenting a “spiral upward” trend. Policy practices from other regions also influence policy adoption behaviors, with local governments tending to imitate cities of similar administrative levels and stronger economic strength. Therefore, actively creating and disseminating exemplary cases of comprehensive smoke-free cities, as well as fully leveraging the demonstrative effect of star cities in tobacco control through research and learning activities, is an important practical path to encourage other cities to adopt comprehensive smoke-free policies.

The negative effects of central policy signals derived from this study differ from conclusions in previous policy formulation or policy innovation diffusion research. Why does a stronger central policy signal decrease the likelihood of local governments adopting comprehensive smoke-free policies? Although the central level in China continuously releases signals for smoke-free environment construction, the policy influence of related documents is limited. Most tobacco control policy documents are issued by health system departments, such as the National Health Commission and the Patriotic Health Campaign Committee, not yet breaking out of the health system to influence cross-sectoral and cross-departmental areas. Moreover, tobacco control policies often appear in forms such as “opinions,” “notices,” and “plans,” dominated by advisory clauses such as “encourage,” “advocate,” and “support,” lacking authoritative enforcement. The study shows that persistently pushing for national-level public place smoke-free legislation remains a core task for future policy advocacy. The interplay among actors involved in smoke-free environment policies, especially the tobacco industry, is complex and merits attention. This study indicates that the greater the economic dependence on tobacco is, the less likely the introduction of comprehensive smoke-free policies will be. China’s tobacco tax data show that national tobacco taxes and profits continue to increase annually, even in cities including Harbin, Changchun, and Xining, which have introduced comprehensive tobacco control policies, with tobacco contributions increasing rather than decreasing. The COVID-19 pandemic has further intensified local governments’ dependence on the tobacco economy. In the process of formulating tobacco control policies, vigilance is crucial regarding the tobacco industry’s use of economic interests as a leverage in negotiations to influence decision makers and ultimately impede the introduction of a comprehensive smoke-free policy.

## 6 Conclusion

This study constructs an analytical framework of factors influencing the adoption of comprehensive smoke-free policies by local governments in China. The framework is based on the three source-flow elements of the multiple streams framework and is tailored through discussions with existing research and the Chinese tobacco control policy scenario. The analytical framework is an adaptation and refinement of the multiple streams framework to the Chinese policy scenario. The study also collects panel data from 36 provincial capitals and other major cities across China from 2013 to 2021. Then, it statistically tests the variables in the analytical framework using logit models with directed dyad-year EHA methodology. The statistical results show that scientific evidence, focal events, media coverage, institutional foundations, horizontal policy diffusion (including learning and imitation mechanisms), central policy signals, and actor influence (including the influence of the health sector, tobacco control groups, and the tobacco system) have significant effects on the adoption of a comprehensive smoke-free policy in a city. Particularly, imitation mechanism, key policy signal, and tobacco industry influence have negative effects, whereas all other variables have positive effects.

### 6.1 Theoretical contributions

On the basis of the multiple streams framework, this study constructs and validates an analytical framework for analyzing the factors influencing the adoption of comprehensive smoke-free policies in key Chinese cities based on the Chinese context. Through EHA, the study validates the core idea of the multisource flow framework (i.e., that policy outcomes are the result of the combined effect of issues, policies, and political flows). When all variables are included in the regression analysis (i.e., the full model), the model has the highest pseudo *R*^2^, suggesting that policy adoption behavior will likely to occur under the combined effects of issues, policies, and political flows. Conversely, differences exist in the positive or negative effects of different factors on policy adoption. Some interesting phenomena are observed. For instance, the national output of tobacco control policy signals did not promote the adoption of a comprehensive smoke-free policy at the local level, and the two were negatively correlated. The study enhances the understanding of policy practices with Chinese characteristics and enriches the applicability and explanatory power of the multiple streams framework to local-level policy processes.

### 6.2 Policy implications

In recent years, health policy has gradually become the focus of domestic public management scholars, but the policy areas of concern are still dominated by “high-attention” areas or emergencies, such as healthcare reform, hospital management, and infectious disease outbreaks, whereas insufficient attention has been paid to “low-attention” policy areas. In the context of China’s epidemiological transition, chronic non-communicable diseases have replaced infectious diseases as the primary risk factor threatening people’s health, and the previous situation of “focusing on treatment but not on prevention” urgently needs to be changed. Thus, research on a large number of non-emergency and low-attention policy areas is conducive to the development of the Healthy China policy and will provide some inspiration for the modernization of the national governance system and governance capacity.

The three source stream framework developed in this study provides a practical guide for advancing tobacco control advocacy. Specifically, in the issue stream, tobacco control advocates can raise policymakers’ awareness of the seriousness of the problem of tobacco control in public places by fully exploring scientific evidence, seizing key policy windows, and strengthening communication and cooperation with the media. In the policy stream, the central government should be encouraged to send clear and binding policy signals. This will encourage local governments to follow and implement policies rather than just “pass through” them; bring into play the roles and functions of different policy subjects in the policy-making process; fully mobilize the health sector and tobacco control groups to participate in the policy-making process; and be wary of the tobacco industry’s negative influence on tobacco control.

### 6.3 Limitations and recommendations

The study has the following limitations that need to be improved in subsequent studies. First, although the quantitative analysis hints at a causal relationship between the influencing factors and policy adoption, it still does not fully open the black box of policy-making. Future studies would benefit from employing other research methods, such case study and process tracking method, should be used in further analyzing the coupling mechanism among those streams. Second, due to data availability, the research focused on the most representative of China’s 36 provincial capitals and above. However, recent years have seen cities such as Zhangjiakou, Qinhuangdao, and Dandong, which are not provincial capitals, also enact comprehensive smoke-free policies. These cities, may have fewer resources for policy advocacy compared with provincial capitals, but they offer valuable lessons on overcoming policy barriers. Their experience warrant attention in future research. Third, the study is conducted in the context of China’s political system, and the explanatory power of the findings for countries with other political systems needs to be further verified in future research.

## Data availability statement

The original contributions presented in the study are included in the article/Supplementary material, further inquiries can be directed to the corresponding author.

## Author contributions

WF: Conceptualization, Methodology, Writing – original draft. BQ: Data curation, Writing – review & editing. XJ: Data curation, Writing – review & editing. SL: Supervision, Validation, Writing – review & editing.
