# Supplementary material for: Identifying factors influencing local governments’ adoption of comprehensive smoke-free policies: an event history analysis based on panel data from 36 key cities in China (2013–2021)
Source: Front Public Health. 2024 Jun 28;12:1397803. doi: 10.3389/fpubh.2024.1397803 (PMC11240852; doi:10.3389/fpubh.2024.1397803)
Supplement: Supplementary file 1 [file Table_1.DOCX]

Supplementary Material

**Table S1** Central Policies on Tobacco control in public places(from 2011 to 2021)

| **No.** | **Policy Title** | **Issuing Authority** | **Dated Issued** | **Link to Original Article** |
| --- | --- | --- | --- | --- |
| 1 | Detailed Rules for the Implementation of the Regulations on the Administration of Sanitation at Public Places (2011) | Ministry of Health (dissolved) | 2011.03.10 | <https://www.pkulaw.com/chl/9e96103155d7a1b2bdfb.html> |
| 2 | The Twelfth Five-Year Plan for National Economic and Social Development of the People's Republic of China | National People's Congress | 2011.03.14 | <https://www.pkulaw.com/chl/603c7a7f2158b52bbdfb.html> |
| 3 | Notice of the State Council on Issuing the Outline for the Development of Chinese Women and the Outline for the Development of Chinese Children (2011) | State Council | 2011.07.30 | <https://www.pkulaw.com/chl/0fe9880c331fcc87bdfb.html> |
| 4 | Notice from 15 departments including the Ministry of Health on the issuance of the "China Chronic Disease Prevention and Treatment Work Plan (2012-2015)" | Ministry of Health (dissolved) | 2012.05.08 | <https://www.pkulaw.com/chl/5238036cfc7a5c8abdfb.html> |
| 5 | Notice of the State Council on Issuing the “Twelfth Five-Year Plan” for the Development of Health Services | State Council | 2012.10.08 | <https://www.pkulaw.com/chl/ec05b018ed67a0c0bdfb.html> |
| 6 | Notice from the Ministry of Industry and Information Technology, the Ministry of Health, the Ministry of Foreign Affairs and others on the issuance of the "China Tobacco Control Plan (2012-2015)" | the Ministry of Industry and Information Technology;Ministry of Health (dissolved);the Ministry of Foreign Affairs | 2012.12 | <https://www.pkulaw.com/chl/246a76d7550dc2eabdfb.html> |
| 7 | Notice from the General Office of the National Health and Family Planning Commission on the issuance of core information on tobacco control and health education | National Health and Family Planning Commission (dissolved) | 2013.08.14 | <https://www.pkulaw.com/chl/dbea2d980528f1d3bdfb.html> |
| 8 | The General Office of the CPC Central Committee and the General Office of the State Council issued the "Notice on Matters Concerning Leading Cadres to Take the Lead in Banning Smoking in Public Places" | General Office of the CPC Central Committee; General Office of the State Council | 2013.12.29 | <https://www.pkulaw.com/chl/1360f7f74de7638bbdfb.html> |
| 9 | Notice from the Ministry of Education on matters related to the ban on smoking in schools at all levels and types across the country | Ministry of Education | 2014.01.17 | <https://www.pkulaw.com/chl/8f6e1c65451e8f4dbdfb.html> |
| 10 | Notice from the General Office of the National Health and Family Planning Commission on further strengthening tobacco control implementation | National Health and Family Planning Commission (dissolved) | 2014.01.26 | <https://www.pkulaw.com/chl/98f7e1ab2737cd69bdfb.html> |
| 11 | Notice of the National Health and Family Planning Commission on Issuing the National Health Literacy Promotion Action Plan (2014-2020) | National Health and Family Planning Commission (dissolved) | 2014.04.15 | <https://www.pkulaw.com/chl/c6fb9a1c9e801719bdfb.html> |
| 12 | Notice of the Central State Organs’ Coordination Leading Group for the Construction of Spiritual Civilization and the Central State Organs’ Patriotic Health Campaign Committee on Launching the “Smoke-free Environment and Civilized Organs” Action of the Central State Organs | Patriotic Health Campaign Committee of the Central State Organizations | 2014.05.07 | <https://www.pkulaw.com/chl/691502bec80a2accbdfb.html> |
| 13 | Notice of the National Patriotic Health Campaign Committee on Issuing the National Sanitary City Standards (2014 Edition) | National Patriotic Health Campaign Committee | 2014.05.15 | <https://www.pkulaw.com/chl/7d7d2fe093e842b6bdfb.html> |
| 14 | Opinions of the State Council on Further Strengthening Patriotic Sanitation Work in the New Era | State Council | 2014.12.23 | <https://www.pkulaw.com/chl/30388bfefb93f20ebdfb.html> |
| 15 | Notice of the National Health and Family Planning Commission and the State Administration of Traditional Chinese Medicine on Issuing an Action Plan to Further Improve Medical Services | National Health and Family Planning Commission (dissolved);National Administration of Traditional Chinese Medicine | 2015.01.12 | <https://www.pkulaw.com/chl/77e669140cdd6431bdfb.html> |
| 16 | Advertising Law of the People's Republic of China (2015 Revision) | Standing Committee of the National People's Congress | 2015.04.24 |  |
| 17 | Notice from the General Office of the National Health and Family Planning Commission, the Secretariat of the Central Civilization Office, the Office of the National Patriotic Association and others on launching the 28th World No Tobacco Day and Healthy China Tour - 2015 Smoke-free Life Theme Publicity and Education Activities | National Health and Family Planning Commission (dissolved);General Office, Central Commission for Guiding Cultural and Ethical Progress;National Patriotic Health Campaign Committee | 2015.05.19 | <https://www.pkulaw.com/chl/b5512d2054c783e3bdfb.html> |
| 18 | Circular of the Ministry of Education on the issuance of the Action Plan for the Improvement of the Management Level of Vocational Colleges and Universities (2015-2018) | Ministry of Education | 2015.08.28 | <https://www.pkulaw.com/chl/f3e352c0b5f46381bdfb.html> |
| 19 | Circular of the National Health and Family Planning Commission, the National Development and Reform Commission and the Ministry of Education on the issuance of a three-year action plan for cancer prevention and treatment in China (2015-2017) | National Health and Family Planning Commission (dissolved);National Development and Reform Commission;Ministry of Education | 2015.09.09 | <https://www.pkulaw.com/chl/e478f4447cab158cbdfb.html> |
| 20 | Detailed Rules for the Implementation of the Regulation on the Administration of Sanitation in Public Places (2016 Amendment PKULAW Version) | National Health and Family Planning Commission (dissolved) | 2016.01.19 | <https://www.pkulaw.com/chl/7c33abdf3adba409bdfb.html> |
| 21 | Outline of the 13th Five-Year Plan for the National Economic and Social Development of the People's Republic of China | National People's Congress | 2016.03.16 | <https://www.pkulaw.com/chl/3fd7b48d6cb8d951bdfb.html> |
| 22 | Outline of the Healthy China 2030 Plan | The Central Committee of the Party;State Council | 2016.10.25 | <https://www.pkulaw.com/chl/a55bb2910f6162efbdfb.html> |
| 23 | Guiding Opinions of the National Health and Family Planning Commission, the Central Propaganda Department and the Ministry of Education on Strengthening Health Promotion and Education | National Health and Family Planning Commission (dissolved);Publicity Department, CPC Central Committee;Ministry of Education | 2016.11.16 | <https://www.pkulaw.com/chl/c241ad10c86cec46bdfb.html> |
| 24 | Circular of the State Council on the Enactment of the 13th Five-Year Plan for Health and Well-Being | State Council | 2016.12.27 | <https://www.pkulaw.com/chl/dbda350786108bbcbdfb.html> |
| 25 | Charity Law of the People's Republic of China | Standing Committee of the National People's Congress | 2016.3.16 |  |
| 26 | Circular of the National Health and Family Planning Commission on the Issuance of the 13th Five-Year Plan for National Health Promotion and Education Work | National Health and Family Planning Commission (dissolved) | 2017.01.11 | <https://www.pkulaw.com/chl/2e3b7135db90fceabdfb.html> |
| 27 | Circular of the General Office of the State Council on the Issuance of the Medium- and Long-Term Plan for the Prevention and Treatment of Chronic Diseases in China (2017-2025) | General Office of the State Council of the People's Republic of China | 2017.01.22 | <https://www.pkulaw.com/chl/00d66935dfee9837bdfb.html> |
| 28 | China unveils 10-year plan for youth development(2016－2025年) | The Central Committee of the Party;State Council | 2017.04 | <https://www.pkulaw.com/chl/c9ba542878e8ad32bdfb.html> |
| 29 | Decision of the First Session of the Thirteenth National People's Congress on the State Council Institutional Reform Proposal | National People's Congress | 2018.03.17 |  |
| 30 | Circular of the General Office of the National Health and Health Commission on the Issuance of the Healthy Dental Action Program (2019-2025) | National Health Commission | 2019.01.31 | <https://www.pkulaw.com/chl/c142130b7ba239c5bdfb.html> |
| 31 | Healthy China Initiative(2019—2030年) | Healthy China Action Promotion Committee | 2019.07.09 | <https://www.pkulaw.com/chl/d65e27553f424b68bdfb.html> |
| 32 | Circular of the National Health and Medical Commission, the Central Propaganda Department and the Ministry of Education on Further Strengthening Tobacco Control for Youth | National Health Commission; Propaganda Department of the Central Committee of the Communist Party of China; Ministry of Education; State Administration of Market Supervision and Administration; National Radio and Television Administration,PRC; State Tobacco Monopoly Administration; Communist Youth League of China; National Women's Federation | 2019.10.29 | <https://www.pkulaw.com/chl/89c890e25c68dfa8bdfb.html> |
| 33 | Basic Healthcare and Health Promotion Law of the People's Republic of China | Standing Committee of the National People's Congress | 2019.12.28 | <https://www.pkulaw.com/chl/22a006a09f3565e4bdfb.html> |
| 34 | Opinions of the State Council on Deepening the Patriotic Hygiene Movement | State Council | 2020.11.14 | <https://www.pkulaw.com/chl/dddebbfe534dafe8bdfb.html> |
| 35 | Opinions of the National Patriotic Health Campaign Committee, the Central Steering Committee for the Construction of Spiritual Civilization, and the Committee for the Promotion of Healthy China's Action on the Promotion of Civilized, Healthy, Green and Environmentally Friendly Lifestyle Activities | National Patriotic Health Committee; Central Commission for Guiding Cultural and Ethical Progress; Healthy China Action Promotion Committee | 2021.01.09 | <https://www.pkulaw.com/chl/f5a2c39c08f62f30bdfb.html> |
| 36 | Circular of the State Council on the issuance of the Program for the Development of Chinese Women and the Program for the Development of Chinese Children (2021) | State Council | 2021.09.08 | <https://www.pkulaw.com/chl/661491589bbbf5a2bdfb.html> |
| 37 | Circular of the National Sanitation Committee on the issuance of the Administrative Measures for the Evaluation of National Sanitary Towns and the Standards for National Sanitary Cities and National Sanitary Counties, and the Standards for National Sanitary Towns and Villages | National Patriotic Health Committee | 2021.12.03 | <https://www.pkulaw.com/chl/0454575a74659e22bdfb.html> |

**Table S2** Evaluation results of smoke-free policies in 36 cities

| **No.** | **City** | **Smoke-free legislation** | **Year** | **Indoor workplaces** | **Indoor Public Places** | | | | | **Public transport** | **Comprehensive smoke-free policy**  **(Y/N)** |
| --- | --- | --- | --- | --- | --- | --- | --- | --- | --- | --- | --- |
|  |  |  |  |  | **Restaurants** | **Bars and entertainment venues** | **Healthcare facilities** | **Government facilities** | **Schools** |  |  |
| 1 | Qingdao | Qingdao Smoking Control Ordinance | 2013 | √ | √ | √ | √ | √ | √ | √ | Y |
| 2 | Shenzhen | Shenzhen Special Economic Zone Smoking Control Ordinance | 2013 | √ | √ | √ | √ | √ | √ | √ | Y |
| 3 | Lanzhou | Lanzhou Public Places Smoking Control Detailed Rules (Provisional) | 2014 | √ | √ | √ | √ | √ | √ | √ | Y |
| 4 | Beijing | Beijing Smoking Control Ordinance | 2014 | √ | √ | √ | √ | √ | √ | √ | Y |
| 5 | Nanning | Nanning Smoking Control Regulations | 2014 | √ | √ | √ | √ | √ | √ | √ | Y |
| 6 | Shanghai | Shanghai Public Places Smoking Control Ordinance | 2016 | √ | √ | √ | √ | √ | √ | √ | Y |
| 7 | Changchun | Changchun Patriotic Health Ordinance | 2016 | √ | √ | √ | √ | √ | √ | √ | Y |
| 8 | Xi’an | Xi’an Smoking Control Measures | 2018 | √ | √ | √ | √ | √ | √ | √ | Y |
| 9 | Hangzhou | Hangzhou Public Places Smoking Control Ordinance | 2018 | √ | √ | √ | √ | √ | √ | √ | Y |
| 10 | Wuhan | Wuhan Smoking Control Ordinance | 2019 | √ | √ | √ | √ | √ | √ | √ | Y |
| 11 | Harbin | Harbin Secondhand Smoke Harm Prevention Ordinance | 2020 | √ | √ | √ | √ | √ | √ | √ | Y |
| 12 | Zhengzhou | Zhengzhou Public Places No Smoking Ordinance | 2020 | √ | √ | √ | √ | √ | √ | √ | Y |
| 13 | Xining | Xining Smoking Control Ordinance (2021 Amendment) | 2021 | √ | √ | √ | √ | √ | √ | √ | Y |
| 14 | Hefei | Hefei Public Places No Smoking Regulations | 1995 | × | × | √ | √ | × | √ | √ | N |
| 15 | Lhasa | Lhasa Patriotic Health Management Regulations Implementation Measures | 2009 | × | × | √ | √ | × | √ | √ | N |
| 16 | Guangzhou | Guangzhou Smoking Control Ordinance | 2010 | √ | × | × | √ | √ | √ | √ | N |
| 17 | Shijiazhuang | Shijiazhuang Public Places No Smoking Regulations | 2010 | × | × | √ | √ | × | √ | √ | N |
| 18 | Tianjin | Tianjin Smoking Control Ordinance | 2012 | √ | × | × | √ | √ | √ | √ | N |
| 19 | Fuzhou | Fuzhou Public Places Smoking Control Ordinance | 2015 | √ | × | × | √ | √ | √ | × | N |
| 20 | Haikou | Haikou Patriotic Health Management Measures | 2016 | × | √ | √ | √ | √ | √ | √ | N |
| 21 | Xiamen | Xiamen Special Economic Zone Regulations on Promoting Social Civility | 2017 | × | × | √ | √ | × | √ | √ | N |
| 22 | Hohhot | Hohhot Regulations on Promoting Civilized Behavior | 2020 | × | √ | × | √ | √ | √ | √ | N |
| 23 | Chongqing | Chongqing Public Places Smoking Control Ordinance | 2020 | √ | × | × | √ | √ | √ | √ | N |
| 24 | Shenyang | Shenyang Smoking Control Ordinance | 2021 | √ | × | × | √ | √ | √ | √ | N |
| 25 | Dalian | Dalian Smoking Control Ordinance | 2021 | √ | × | × | √ | √ | √ | √ | N |
| 26 | Nanchang | Nanchang Public Places No Smoking Temporary Regulations | 1995 | × | × | × | × | × | × | × | N |
| 27 | Jinan | Jinan Public Places No Smoking Regulations | 1996 | × | × | × | × | × | × | × | N |
| 28 | Chengdu | Chengdu Public Places No Smoking Regulations | 1996 | × | × | × | √ | × | √ | × | N |
| 29 | Nanjing | Nanjing Public Places No Smoking Temporary Regulations | 1997 | × | × | × | √ | × | √ | √ | N |
| 30 | Urumqi | Urumqi Public Places No Smoking Regulations | 1998 | × | × | × | × | × | × | × | N |
| 31 | Kunming | Kunming Public Places No Smoking Management Measures | 2002 | × | × | × | × | × | × | × | N |
| 32 | Ningbo | Ningbo Patriotic Health Ordinance | 2006 | × | × | × | √ | × | √ | × | N |
| 33 | Yinchuan | Yinchuan Public Places Smoking Control Ordinance | 2009 | × | × | × | √ | × | √ | × | N |
| 34 | Changsha | Changsha Regulations on Promoting Civilized Behavior | 2019 | × | × | × | × | × | × | √ | N |
| 35 | Guiyang | Guiyang Public Places No Smoking Temporary Regulations | 2020 | × | × | × | × | × | × | × | N |
| 36 | Taiyuan | No relevant regulations | / | × | × | × | × | × | × | × | N |
